# Emerging frontiers in epigenetic-targeted therapeutics for pediatric neuroblastoma

**DOI:** 10.3389/fimmu.2025.1637626

**Published:** 2025-07-25

**Authors:** Xiaokang Wang, Gengrui Xu, Hongyan Ma, Xiaoyan Deng, Guiping Ma

**Affiliations:** ^1^ Department of Pharmacy, Shenzhen Longhua District Central Hospital, Affiliated Longhua Hospital of Shenzhen University, Shenzhen, China; ^2^ Center of Community Health Service Management, Shenzhen Longhua District Central Hospital, Shenzhen, China; ^3^ Beijing University of Chinese Medicine Affiliated Shenzhen Hospital (Longgang), Shenzhen, China

**Keywords:** immunologically cold, small-molecule targeted agents, epigenetic regulation, pediatric neuroblastoma, therapeutic strategies

## Abstract

Neuroblastoma (NB), the most prevalent extracranial solid malignancy in children, poses significant therapeutic challenges, particularly concerning high-risk subtypes characterized by an immunologically “cold” phenotype. These tumors evade immune surveillance through mechanisms such as impaired antigen presentation and immunosuppressive microenvironment formation. Despite the incorporation of immunotherapies (*e.g.*, GD2 monoclonal antibodies) into international clinical guidelines, the 5-year survival rate of patients with NB persistently remains lower than 50%. Small-molecule targeted agents, distinguished by their low molecular weight and superior chemical stability, offer advantages over macromolecular antibody therapies by effectively penetrating cell membranes to engage intracellular targets. Epigenetic regulation, a DNA sequence-independent gene expression modulation system, plays a pivotal role in cell fate determination *via* dynamic DNA methylation, histone covalent modifications, chromatin spatial reorganization, and non-coding RNA-mediated pathways. Emerging evidence has highlighted a strong correlation between epigenetic dysregulation and NB progression, establishing a molecular rationale for novel therapeutic strategies. Current epigenetic research in NB primarily focuses on histone deacetylase inhibitors and DNA methyltransferase inhibitors. These drugs exhibit unique translational potential because of their accelerated development timelines and cost-effective production, significantly enhancing therapeutic accessibility. This review systematically examines the current landscape of epigenetic modulators in NB treatment and discusses their transformative potential in improving outcomes for high-risk patients with NB.

## 1 Introduction

Neuroblastoma (NB), the most prevalent extracranial solid tumor in children, accounts for 8%–10% of pediatric malignancies, is often termed the “king of childhood cancers” because of its aggressive behavior and dismal prognosis (1). Originating from neural crest-derived sympathetic ganglion cells, NB predominantly arises in the adrenal medulla (55%–60% of cases) and paravertebral sympathetic chains and less frequently arises in the mediastinum (20%) and pelvis (15%) (2, 3). Epidemiological studies reported an annual incidence of 6–8 cases per million children, highlighting its significant clinical variability and challenging therapeutic landscape (4).

Contemporary NB management confronts three principal challenges: diagnostic delays (60% of patients present with distant metastases to bone marrow, skeletal systems, or liver at diagnosis, resulting in persistently stagnant 5-year survival rates of 40%–50% in high-risk cohorts (5)); therapeutic limitations (even with multimodal intensive regimens combining surgical resection, high-dose chemotherapy, autologous stem cell transplantation, and radiotherapy, survival outcomes have not significantly improved (6)); and immune evasion mechanisms (including tumor microenvironment alterations such as MHC class I downregulation and PD-L1 overexpression, which compromise the efficacy of GD2 monoclonal antibody combined with retinoic acid immunotherapy (7)).

Molecular profiling has identified pivotal oncogenic drivers such as MYCN amplification (25%–30%) and ALK mutations (8%–10%) (8, 9). Although these discoveries have refined risk stratification systems, their translational potential remains unrealized. Consequently, the development of novel therapies targeting tumor stem cell eradication and epigenetic regulation has become an urgent priority in contemporary research.

Epigenetic therapeutics represent a promising intervention strategy to overcome chemoresistance and relapse in high-risk NB (HR-NB) (10). This approach focuses on modulating epigenetic regulators, which are critical functional proteins orchestrating dynamic chromatin remodeling (11). These regulators mediate multilayered control through DNA methylation (5mC/5hmC), histone modification (*e.g.*, H3 lysine 27 methylation [H3K27me3], H3 lysine 9 acetylation [H3K9ac], and non-coding RNA (ncRNAs) networks (*e.g.*, lncRNAs, miRNAs), enabling spatiotemporal gene expression regulation without altering DNA sequences (12). The inherent reversibility of epigenetic modifications renders them ideal therapeutic targets, with epigenetic drug targets constituting 18.7% of all cancer therapeutic targets (12). The key advantages of epigenetic drugs lie in their multipathway synergy enabling the coordinated modulation of MYCN signaling and p53 restoration through single-target interventions, bidirectional transcriptional control that simultaneously suppresses oncogene hyperactivation (*e.g.*, ALK, PHOX2B) and reactivates epigenetically silenced tumor suppressors (e.g., CASZ1, CLU), and heritable chromatin remodeling effects ensuring sustained therapeutic outcomes through the stable transmission of modified chromatin states across cell divisions.

Recent genomic analyses have positioned NB as a biologically distinct solid tumor characterized by a remarkably low somatic mutation burden and the absence of dominant driver genes. This recognition has catalyzed a paradigm shift emphasizing epigenetic dysregulation as potentially central to NB pathogenesis. Whole-genome analyses have identified three hallmark epigenetic aberrations: DNA methylation landscape remodeling featuring genome-wide hypomethylation coupled with promoter-specific hypermethylation (*e.g.*, >80% methylation at HOX gene clusters); histone modification imbalances exemplified by H3K27me3 depletion (observed in 62% of high-risk cases) and abnormal H3K4me3 accumulation; and chromatin remodeler dysfunction, including frequent subunit deletions in SWI/SNF complexes (up to 40%). Despite identifying characteristic alterations such as PRC2 overexpression and TET enzyme inactivation, clinically validated epigenetic biomarkers remain elusive for diagnostic or prognostic applications (13).

This comprehensive review systematically examines the molecular foundations of epigenetic dysregulation in NB, the clinical translation of existing epigenetic therapeutics, and rational combination therapy strategies.

## 2 Cellular origins and transformation mechanisms of NB

**Figure 1:**
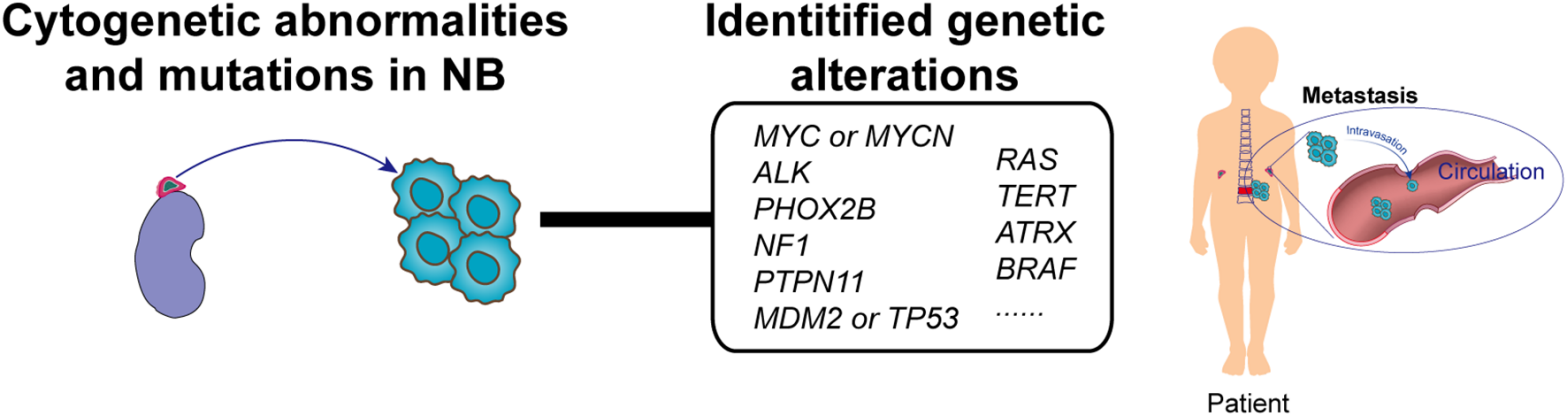
NB pathogenesis and key genetic alterations. Adrenergic lineage cells represent the predominant cellular origin of NB. NB pathogenesis is driven by the multilayered interplay of genomic aberrations. At the genomic level, recurrent somatic alterations include MYCN amplification, activating ALK mutations, and ATRX deletions.

As the most prevalent pediatric extracranial solid tumor, NB arises from malignant transformation during the sympathetic–adrenal lineage differentiation of neural crest cells (NCCs). Tumorigenesis is initiated when NCC-derived chromaffin cell precursors undergo developmental arrest at critical differentiation checkpoints during the seventh gestational week, coinciding with their migration to the adrenal primordium. Accumulating evidence positions adrenergic lineage cells as the principal cellular origin of NB, with single-cell transcriptomic profiling demonstrating striking transcriptional congruence between NB tumor cells and fetal adrenal chromaffin progenitors (**Figure 1**). This molecular mimicry, preserved through the malignant reprogramming of developmental pathways, provides compelling evidence for the adrenal chromaffin origin hypothesis while revealing critical vulnerabilities in NB’s epigenomic regulatory architecture.

NB pathogenesis is driven by the multilayered interplay of genomic and epigenetic aberrations. Genomically, recurrent somatic alterations include MYCN amplification (20% of cases, associated with 5-year survival rates of <50%), activating ALK F1174L mutations (8%), and ATRX deletions (11% in adolescents), with NF1 loss-of-function mutations synergizing with MYCN to drive tumorigenesis (14). Emerging evidence has further established clinical correlations of PHOX2B, TP53, RAS, and BRAF mutations with NB progression (**Figure 1**) (15, 16). Chromosomal instability manifests through pathognomonic chromothripsis events, which are detected in 19% of MYCN-amplified tumors. Epigenetically, coordinated dysregulation is typified by DNA methylation paradox (genome-wide hypomethylation coexisting with CpG island hypermethylation), EZH2 overexpression-mediated histone modification imbalance, and ncRNA networks governing proliferation-apoptosis homeostasis. These multilayered mechanisms converge to reshape developmental checkpoints and survival pathways, establishing NB’s unique oncogenic landscape.

## 3 The role of epigenetics in NB therapy

### 3.1 DNA methylation regulatory networks and therapeutic targeting

**Figure 2:**
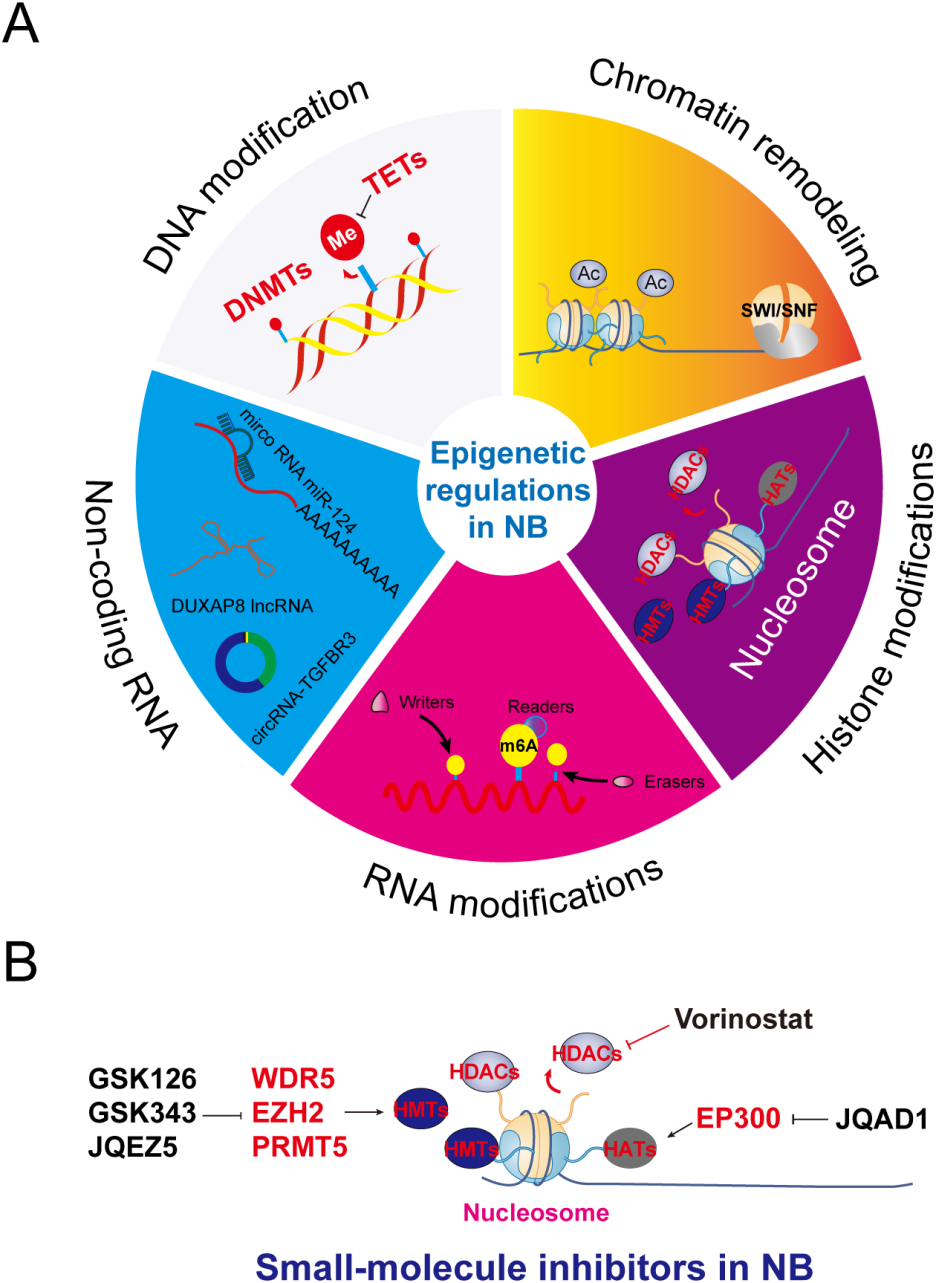
Epigenetic mechanisms and key examples of widely studied modifications and their modifying enzymes. **(A)** DNA modifications, chromatin remodeling, histone modifications, RNA modifications, and ncRNA-based regulation constitute the core content of epigenetics, being responsible for passing on heritable variations of genetic information independently of the DNA sequence. **(B)** Mechanistic schematic of epigenetic-targeting small-molecule inhibitors in NB.

The dynamic equilibrium of DNA methylation is orchestrated by the antagonistic interplay between DNA methyltransferases (DNMTs) and ten-eleven translocation (TET) dioxygenases. Emerging pan-cancer analyses have revealed divergent TET family expression patterns: TET1 is transcriptionally silenced in 63% of hepatocellular carcinomas, whereas TET2 is mutated in 17% of gliomas (TCGA data). NB-specific epigenetic studies (17–19) identified TET3 as a potential prognostic biomarker, with its expression inversely correlating with the mitotic–karyorrhexis index. Elevated TET3 expression is correlated with improved 5-year survival (42%), and the tumor-suppressive role of TET3 is mechanistically linked to the 5mC hydroxylation-mediated maintenance of open chromatin states at neurodifferentiation-associated genes (*e.g.*, PHOX2B). Contrastingly, TET1 drives oncogenesis through β2-adrenergic receptor pathway activation, inducing cAMP–PKA signaling that stabilizes MYCN protein (3.2-fold extended half-life). This isoform additionally partners with the histone demethylase KDM6B to form a transcriptional activation complex promoting tumor progression (20). The development of isoform-selective TET1 inhibitors and TET3 agonists represents a promising frontier for epigenetic therapy in NB (**Figure 2A**).

Pioneering work by the Alaminos group (21) first elucidated the critical association among CpG island hypermethylation, MYCN amplification, and poor clinical outcomes in NB. Therapeutically, DNA methyltransferase inhibitors (*e.g.*, decitabine) demonstrate dual efficacy, exhibiting standalone antiproliferative effects while synergistically enhancing conventional chemotherapeutics, a paradigm validated across multiple pediatric NB clinical trials. Notably, cisplatin-resistant models and high-risk NB subtypes exhibit marked upregulation of DNMT3A/B isoforms. Selective targeting of DNMT3B with nanaomycin A induces tumor-selective apoptosis through global methylation reduction (22), revealing a vulnerability in treatment-refractory disease. With the advancement of genome-wide methylation profiling technologies, high-resolution methylation mapping promises to resolve long-standing debates about the cellular origins of NB while informing precision epigenetic therapies (23).

### 3.2 Histone modification circuitry and therapeutic vulnerabilities in NB

Emerging evidence positions histone deacetylases (HDAC8/HDAC10) as central regulators of NB proliferation, differentiation, and chemosensitivity. Preclinical studies demonstrated that pan-HDAC inhibitors such as vorinostat suppress tumor growth through dual mechanisms: cell cycle arrest (G1 phase accumulation) and intrinsic apoptosis activation (caspase-3 cleavage) (24). These agents synergize with conventional chemotherapy or radiation, achieving enhanced tumor regression in xenograft models. Second-generation inhibitors (*e.g.*, panobinostat, romidepsin) further exhibit blood–brain barrier penetration efficacy and display promise in central nervous system-metastasized NB subtypes (25), with six active HDAC-targeted clinical trials currently recruiting pediatric patients with NB. Paradoxically, although HDAC inhibition broadly suppresses oncogenic programs, context-dependent activation of differentiation pathways could underlie its therapeutic duality.

Beyond HDAC targeting, multilayered histone methylation networks involving WDR5 (H3K79me modulator), EZH2 (H3K27me3 writer), and PRTM5 (H3K4me3 eraser) orchestrate NB plasticity. WDR5–MYC complexes drive super-enhancer formation at oncogenic loci, whereas EZH2-mediated PRC2 activation silences tumor suppressors such as CLU and CADM1. PRMT5-centric arginine methylation sustains spliceosome integrity in MYCN-amplified cells. Pharmacological disruption of these nodes (*e.g.*, EZH2 inhibitor tazemetostat, PRMT5 inhibitor GSK3326595) induces differentiation and reverses chemoresistance in preclinical models (26). These findings collectively map NB’s epigenetic vulnerabilities, facilitating the development of novel combinatorial strategies that simultaneously target histone-modifying enzymes and lineage-specific oncogenic drivers (**Figure 2B**).

The precise equilibrium between histone acetylation and deacetylation serves as a master epigenetic switch governing transcriptional plasticity, with its dysregulation representing a hallmark of tumorigenesis. In high-risk NB, HDAC8 and HDAC10 exhibit pathologic overexpression (27, 28), establishing them as actionable targets. Pharmacologic HDAC inhibition both suppresses tumor proliferation and chemosensitizes resistant cells to doxorubicin, potentially overcoming treatment barriers. Preclinical studies identified valproic acid as a broad-spectrum HDAC inhibitor capable of inducing a triad of effects: proliferation arrest, mitochondrial apoptosis, and neural differentiation.

Vorinostat, the first FDA-approved pan-HDAC inhibitor, demonstrates mechanistically distinct antitumor activity in NB. This drug triggers G2/M phase blockade through CDK1/cyclin B dysregulation and activates intrinsic apoptosis *via* Bim/PUMA transcriptional induction. Clinical translation has revealed striking efficacy. Combined with ^131^I-metaiodobenzylguanidine (MIBG), vorinostat elevates objective response rates in relapsed/refractory NB from 14% to 32% and extends median progression-free survival by 5.8 months (ASCO 2021 data) (29). Synergistic potential was further evidenced by its combined use with panobinostat, which extended survival in TH-MYCN transgenic mice by 62%, and with GD2-targeted immunotherapy, in which co-treatment enhanced antibody-dependent cellular cytotoxicity (30). These multimodal regimens exemplify the evolving paradigm of epigenetic-immune interplay in NB precision medicine.

Targeting SWI/SNF complex dysregulation represents a therapeutic mechanism in NB. Approximately 25% of human cancers harbor mutations in genes encoding mammalian SWI/SNF (mSWI/SNF) chromatin remodeling complexes (31). Core components include ATPase subunits (SMARCA4/BRG1 and SMARCA2/BRM) and structural subunits (*e.g.*, SMARCC1/2, SMARCD1/2/3) (32). Mechanistically, SMARCB1 mutations induce the mislocalization of mSWI/SNF complexes at gene promoter regions accompanied by RNA polymerase II dysfunction and altered H3K27ac signatures. In NB, mutations in the ARID1A/ARID1B subunits of the SWI/SNF chromatin remodeling complex promote tumor progression and correlate with poor prognosis.

Emerging evidence has revealed the histone acetyltransferase EP300 as an epigenetic linchpin in NB pathogenesis, particularly through MYCN transcriptional regulation (33). Mechanistically, EP300 acetylates histones at MYCN super-enhancer regions, thereby sustaining oncogene addiction in MYCN-amplified tumors. Pioneering work by Durbin et al. (33) developed JQAD1, a PROTAC-based EP300 degrader that achieves tumor-selective depletion (90% reduction at 100 nM) through VHL E3 ligase recruitment. This agent induces rapid apoptosis (caspase-3 activation within 8 h) in MYCN-driven models while demonstrating exceptional safety margins (normal cell viability > 85%), establishing targeted protein degradation as a breakthrough paradigm.

Paralleling these advances, the histone methyltransferase EZH2 exhibits co-operative oncogenesis in MYCN-amplified NB. MYCN transcriptionally upregulates EZH2 (34) while physically interacting with its N-terminal domain to stabilize MYCN protein through PRC2-catalytic-independent mechanisms (35), creating a feed-forward malignancy loop. Pharmacologic disruption using catalytic inhibitors (GSK126, IC_50_ = 6 nM; JQEZ5, IC_50_ = 8 nM) induces tumor regression in orthotopic models (36), with current clinical efforts exploring combination regimens with BET inhibitors.

Despite these successes, structural biology limitations persist, as the domain structure has been resolved for fewer than 30% of epigenetic regulators, hampering the rational design of isoform-selective agents. Next-generation approaches currently prioritize covalent EZH2 inhibitors (*e.g.*, MS1943) and dual EZH2/HDAC degraders to overcome compensatory resistance mechanisms, although tumor-selective delivery remains a critical translational barrier.

### 3.3 ncRNAs networks and therapeutic opportunities in NB

ncRNAs have emerged as master epigenetic regulators governing NB tumorigenesis, with distinct subclasses, namely miRNAs, lncRNAs, and circular RNAs (circRNAs), orchestrating malignant hallmarks through multilayered gene regulatory networks (37–39). Clinically, circulating miRNAs exhibit diagnostic potential through their exosome-mediated intercellular communication and stability in biofluids (40). For instance, miR-124 acts as a tumor suppressor by reversing therapy resistance in mesenchymal-type NB cells, and its targeted upregulation using PP121 (a tyrosine/PI3K kinase inhibitor) synergizes with BDNF-activated bufalin to induce neural differentiation and apoptosis (41, 42). This exemplifies the therapeutic potential of miRNA modulation in overcoming NB chemoresistance.

The lncRNA landscape reveals equally critical oncogenic drivers (37). DUXAP8, which is overexpressed in > 60% of high-risk NB tumors, accelerates tumor progression *via* dual mechanisms: sequestering miR-29 to derepress NOL4L-mediated cell cycle activation and enhancing metastatic potential through TWIST1 stabilization. CRISPR-mediated DUXAP8 silencing reduces xenograft tumor growth by 72%, validating its therapeutic candidacy.

The discovery of circRNAs has unveiled a new regulatory layer in NB pathogenesis, with MYCN amplification profoundly altering circRNA landscapes (43). Comparative deep sequencing analysis of five MYCN-amplified NB tumors versus matched normal tissues revealed 2242 significantly downregulated circRNAs, among which three tumor-suppressive circRNAs exhibited particularly promising therapeutic potential. Specifically, circTBC1D4 functions as a molecular sponge for oncogenic miR-21, thereby de-repressing PDCD4 expression and restoring apoptosis sensitivity in treatment-resistant cells. circNAALAD2 directly interacts with the PHLPP2 phosphatase to suppress AKT hyperphosphorylation, effectively inhibiting PI3K-driven survival pathways. circTGFBR3 structurally stabilizes the AXIN1–APC destruction complex, leading to β-catenin degradation and Wnt pathway suppression (44).

The primary mechanisms and functions of these epigenetic drugs in NB treatment are summarized in **Table 1**. These agents demonstrate multitarget regulatory capabilities in NB disease progression by modulating key nodes including apoptosis, proliferation, and epigenetic modifications, offering multifaceted mechanisms and potential therapeutic targets for neuroblastoma therapy. However, few epigenetic drugs have advanced to clinical trial phases.

**Table 1:** Epigenetic drugs in NB.

Drug name	Functions *in vivo*	References
*m*-Carboxycinnamic acid bishydroxamide	Apoptotic cell death	(45, 46)
MS-275	Restores the p53 tumor-repressor function	(47)
BL1521	Inhibits proliferation and induces apoptosis; cell cycle arrest and differentiation	(48, 49)
Trichostatin A	Increases cell viability and antioxidant capacity	(50)
Romidepsin	Controls growth and induces apoptosis	(25)
3-Deazaneplanocin A	Increases tumor suppressors	(51)
GSK126/GSK343, JQEZ5	Inhibits cell differentiation and gene expression regulator	(36, 51)
Tazemetostat	Combats NB immune evasion	(52)
Valemetostat	Reactivates tumor suppressor genes	(53)
EP300, JQAD1	Induces apoptosis	(33)
Valproic acid	Increases proliferation and induces apoptosis	(54)
Vorinostat	Inhibits cellular growth	(24, 55)
Decitabine	Inhibits cellular growth	(56)
Nanaomycin A	Induces apoptosis	(57)
circRNA-TBC1D4, circRNA-NAALAD2, circRNA-TGFBR3	Inhibits miR-21 related pathways and suppresses proliferation, migration and invasion	(44)

Text in red color in the table represents Phase I clinical trials.

Although the number of active clinical trials for epigenetic modifiers in NB remains limited, a substantial pool of potential novel epigenetic targets awaits exploration. The epigenetic regulatory genes with therapeutic potential for NB identified in current preclinical studies (**Table 2**) will facilitate the development of new compounds (epigenetic drugs).

**Table 2:** Potential epigenetic targets in NB.

Target gene	Functions *in vivo*	Reference
NSD1	Cell proliferation/inhibition of cellular growth	(51, 58)
PRMT5	Cell proliferation/survival	(59)
KDM1A	Cell proliferation/invasion	(60, 61)
JMJD1A	Migration/invasion	(62)
JARID1B	Invasion/chemoresistance	(63)
HDAC2	Increases proliferation/survival	(64)
HDAC5	Blocks differentiation/induces proliferation	(65)
HDAC6	Regulates cell survival	(66, 67)
HDAC11	Regulates cell survival	(68, 69)
SIRT2	Increases proliferation	(70)

Light blue shading in the table denotes histone methyl-transferases. Orange shading in the table denotes histone deacetylases. Dark blue shading in the table denotes histone demethylases.

## 4 Diversified development in NB targeted therapy

Diversified clinical advances have been achieved in NB targeted therapy. Antibody-based immunotherapies (*e.g.*, dinutuximab beta, naxitamab) are established frontline interventions, whereas small-molecule targeted agents offer advantages such as low molecular weight, oral bioavailability, and favorable cost-effectiveness. Eflornithine remains the only approved oral maintenance therapy, complementing preclinical-stage epigenetic modulators such as HDAC inhibitors. Mechanistic insights into NB pathogenesis have propelled the development of novel compounds such as IBL-302 (BRD4-targeting PROTAC) and APG-115 (MDM2 degrader), which are currently undergoing Phase I/II clinical trials after producing breakthrough efficacy in MYCN-driven models.

Notably, NB’s pronounced genomic heterogeneity poses significant therapeutic hurdles, as 40% of relapsed tumors exhibit ALK/RAS pathway co-activation. Future strategies require integrating multiomics platforms (single-cell epigenomics, spatial proteomics) to identify druggable targets, thereby addressing key obstacles in developing NB-targeted small molecules through precision target validation and pharmacological exploitation of genomic vulnerabilities.

Advanced ncRNA delivery systems have demonstrated transformative therapeutic potential. Specifically, nanoparticle encapsulation significantly enhances ncRNA stability and bioavailability, whereas naturally derived exosomes, with their inherent low immunogenicity and blood–brain barrier penetrance, enable targeted ncRNA delivery to NB cells without triggering immune responses. The field’s future development will strategically focus on three key directions: molecular therapeutics featuring MYCN/ALK inhibitors (lorlatinib), CDK4/6 inhibitors (ribociclib), and TRK inhibitors (entrectinib/larotrectinib) currently in clinical trials; advanced delivery platforms utilizing CRISPR-modified small extracellular vesicles that establish pre-metastatic niches through precision immune cell priming (71); and regimen optimization *via* chronologically coordinated combination therapies (*e.g.*, ^131^I-MIBG radiotherapy with GD2-targeted immunotherapy or CAR-T regimens) to simultaneously enhance therapeutic efficacy and reduce long-term complications.
